# Childhood Circumstances and Mental Health in Old Age: A Life Course Survey in China

**DOI:** 10.3390/ijerph18126420

**Published:** 2021-06-14

**Authors:** Huoyun Zhu, Mengting Liao

**Affiliations:** School of Public Administration and Emergency Management, Jinan University, Guangzhou 510632, China; turbomontine@163.com

**Keywords:** population ageing, childhood circumstances, mental health, life course, active ageing

## Abstract

Current evidence and research of the life course approach on the association between life experiences and health in old age are fragmentary. This paper empirically examines the “long arm” effect of the childhood circumstances on mental health in later life using a large longitudinal dataset (CHARLS) conducted in 2014 and 2015. We operationalize the childhood circumstances as family economic conditions, community environment, and peer network to include the meaningful content and understand their interaction. The SEM results indicate that effects of those factors contributing to older people’s mental health are unequal and vary among age groups and genders. Of those, peer network in childhood determines to a large extent the mental health through the whole life course, while economic conditions and community environment are weakly associated with mental health. Furthermore, we find a distinct interaction mechanism linking those variables. The peer network completely mediates the effect of the community environment on the mental health of older adults and has a partial mediating effect on the economic conditions. Those findings suggest that social policies aimed at promoting older people’s mental health in the context of the active ageing and health ageing strategy should go beyond the old age stage and target social conditions early in childhood.

## 1. Introduction

Population ageing is the most remarkable global population trend in this century. Historically low levels of fertility combined with increased longevity ensure that the population virtually throughout the world is growing older. In 2018, it was the first time in human history that the population aged 65 and over outnumbered children under five years worldwide. Older people aged 65 and over are projected to more than double between 2019 and 2050 to 1.5 billion, which will be more than adolescents and youth aged 15 and 24 years (1.3 billion) [1]. As the country with the largest population of older adults, while still a developing one, China is confronted with considerable challenges in developing an active ageing strategy to improve older people’s life quality. Quality of life, especially in terms of older adults, is intensively linked to health status, which was included as one of three elements of the active ageing strategy by the World Health Organization (WHO). The WHO has initiated a Global Strategy and Action Plan for Ageing and Health for 2016–2020 [2], followed by its continuation with the Decade of Healthy Ageing 2020–2030 [3]. Accordingly, the Chinese central government issued a national strategy, ‘health China 2030’ [4], in 2016, aimed at promoting the national population quality and serving the social and economic development better, which is one part of the Sustainable Development Goals (SDGs) proposed by the UN. As many as 13 development goals have been set out in this national strategy for the next 15 years. For example, the life expectancy at birth is planned to increase to 79 years in 2030.

How to reach those goals is the following challenge for policymakers. Although social care services and medical treatment are essential to older people, more and more empirical evidence has suggested that the intervention measures focusing on the earlier life stage would make much better outcomes [5,6]. Closely linked to the population ageing trend is an epidemiological transition involving a progressive shift from maternal, child, and communicable diseases to chronic noncommunicable disorders [7]. The majority of health problems of the older population are associated with chronic diseases such as hypertension, diabetes, dementia. Many of them are accumulated across the life course. This implies that they can be successfully delayed or prevented by healthier daily behaviors in earlier life [8]. A bounty of scientific evidence shows that social factors including maternal nutrition, socioeconomic conditions, physical inactivity progressively contribute to non-communicable diseases with age compared to the biological ones [9]. The Secretariat of the WHO has accordingly published an action plan named “Multisectional action for a life course approach to healthy ageing: draft global strategy and plan of action on ageing and health” on the 69th World Health Assembly in 2016. This plan states that since many of the disorders of older age are preventable, and many of their determinants begin earlier in life, some measures needed to take for the prevention of disease and declines in capacity. Such as, at younger ages, priority will be on preventing the common noncommunicable diseases by enabling physical activity and good nutrition, avoiding tobacco, and fostering the responsible use of alcohol. However, current evidence and research are fragmentary, additional empirical findings are called for to comprehensively understand the relationship between earlier life experience and health in old age, particularly in less developed countries where older people have experienced extremely poor living conditions during their childhood.

This study aims at investigating the direct linking mechanism between life experience during childhood and the mental health in later life by incorporating multiple indicators of living circumstances. A high-quality dataset from a national life course survey conducted by Peking University is utilized by applying the Structural Equation Model (SEM) to explore this relationship. The following part of the article consists of four sections. First, a literature review on the factors of the health of older people with focus on those related to childhood from the life course perspective is put forward. The second part introduces the research design encompassing theoretical framework, data sources, variable measurement, and quantitative methods. Then, the results of the descriptive and model analysis are presented in the third section. Subgroup comparisons are made for age and gender heterogeneity in this part. On the one hand, we are concerned about the significant health disparity between genders, which is a globally prevalent phenomenon and well accepted by the academic community. In China, this difference is particularly pronounced due to the influence of the traditional gender role norms and social policies preferring men. On the other hand, there is a significant heterogeneity in health status among older adults. Multiple comparison by age cohort helps to examine the varied influences of childhood living circumstances on mental health in later life. Last, some vital findings are highlighted based on previous studies in the discussion section followed by a brief conclusion.

## 2. Literature Review

### 2.1. Factors That Contribute to the Health of Older People

The health of older people is influenced by a series of factors which interact with each other. Those factors can be sorted into two parts according to the timeline: current and historical factors. Earlier studies focus on the direct influence of the current living conditions. Older people with better living conditions and habits are more likely to be in better health [10,11]. He [12] found that the higher the socioeconomic status (such as education, income, and occupation) of older people, the better their health status. Chen [13] found that older adults with their children’s support and social security are more likely to have better health. In addition, lifestyles such as dietary habits, physical activity, and living arrangements are also strongly associated with the health of older adults [14].

The second approach derives from the life course theory emerging in the 1970s and views that the health in old age is more the result of the accumulation of the early life period other than a discrete one-time event [15]. A person’s experience in the early life period has largely determined the health in their later life. Since Glenn Elder introduced the concept of “life course”, it has been widely used as a theory, a perspective, or a paradigm in social and behavioral sciences [16]. Increasing scholars have focused on the influence of early life on middle and later age, and a lot of research has emerged that examines the impact of childhood experiences on health in later life based on a life course perspective. Studies based on this perspective can be further classified in a broad sense into two categories.

The first category is from the perspective of biomedicine or psychology. This approach emphasizes that childhood is the initial stage of the personality growth and development which, to a considerable extent, determines the health status in adulthood and even in old age [17,18]. It has been confirmed that the adverse events experienced in childhood may influence the risk of individual health problems by affecting brain development [5]. However, the health effects of such events are not unidirectional. Taking the experience of starvation as an example, if people suffered from starvation in childhood, the incidence of heart disease, peripheral arterial disease, and diabetes will increase in later life. However, an alternative theory such as the Hormesis exists, and results from experiments on a number of animals and humans suggest that caloric restriction or fasting is associated with improvements in individual health, such as slower ageing and lower prevalence of diabetes and coronary artery disease diagnoses [19,20].

The other is based on the sociological perspective. Scholars take parents’ occupation, income, education, etc., as the measurement indicators of the SES (socioeconomic status) in childhood, and find that better SES in childhood will have a positive impact on the health of the elderly, while lower SES may lead to serious health problems in middle-aged and older people [21,22,23]. For example, lower family income in childhood and lower education of parents, will not only increase the morbidity and mortality in old age [24], but also have a significant relationship with cognitive impairment, physical dysfunction, and depression of older people [25,26]. Some scholars also pay attention to the relationship between the adverse experience referring to the community environment beyond individual capacity in childhood and older people’s health. Studies have shown that the adversity people experienced in childhood has a long-term negative impact on their mental health [27]. Children who experienced poverty, starvation, family disintegration, and abuse in the early stage are at higher risk of poorer health in old age than those who did not. The misfortunes experienced in childhood have a cumulative effect on their health in later years. The more unfortunate events experienced in childhood, the worse their health in old age [6]. Individuals who have experienced childhood adversity such as bullying, physical or mental abuse, and parents’ death are 2.78 times more likely to suffer from mental illness than their counterparts [28]. In addition, the risk of mental depression in these groups in their later years will also significantly increased [5,29].

### 2.2. Influence Mechanism of Childhood Factors on Health in Old Age

According to the existing research, there are two different views on how childhood life experiences affect health in old age. One is the direct mechanism of action. The fetal origins hypothesis proposed by Barker suggests that the fetus and infant are critical periods of growth and development, and events experienced during this period can have a significant impact on future health. However, the impact of the environment on future health is complex. The concept of mismatch being referred to in the Barker hypothesis, focuses on the impact of the degree to which the expected fetal survival environment differs from reality on health outcomes. To adequately adapt to environmental challenges, such as malnutrition or stress, transmitted by the mother, the fetus or young child alters gene expression through epigenetic programming to produce the most environmentally appropriate phenotype in the offspring, and these challenges persist into adulthood. So, even if the fetus experiences malnutrition in utero, if this matches well with a malnourished environment outside the womb, it may provide a form of protection for its development and growth, allowing it to develop well. However, a mismatch between the prenatal and postnatal environment can lead to a greater risk of disease [30,31,32]. According to the above hypothesis, the critical period model of life course theory puts forward that negative events in childhood, a critical stage of development, have a great probability of fundamentally changing health in later life. Under this model, the life experiences in childhood are independent of the changes in adulthood and have a direct impact on the health in later life [33]. Cao and Du [23] prove that the early family economic status has a direct impact on the health of the elderly. The better the early family economic status, the higher the incidence of the elderly’s health.

The other, on the contrary, is the indirect way. The pathway model of life course theory suggests that childhood living conditions influence health in old age through adult life since the good living conditions in adulthood can moderate to some extent the negative health effects of early adverse life experiences and further contribute to health in later life [34], which emphasizes the mediating role of adulthood in the relationship between living conditions in childhood and health in later life. Shi and Yang [29] found that the educational and economic status of an individual in adulthood will play a regulatory role in exploring the effects of childhood abuse on the mental health of the elderly.

In general, studies have yielded rich insights, but there is still room for further investigation. On the one hand, there are relatively few studies focusing on China, a typical developing society. Compared to developed countries where the society, economy, politics, and culture develop gradually and people in which can easily adapt to such an environment, in contrast, developing countries experience a dramatic transition within a shorter period and people in which would experience more varied socioeconomic environments at each life stage than their counterparts in developed ones, which certainly has a significant impact on mental health. This trend is expected to continue in the future. On the other hand, most literature exclusively focuses a single factor such as material security to the health in later life, neglecting other dimensions. It is worthwhile to take more factors into consideration in a full model to investigate their interaction and influence mechanism. In this study, the earlier experience during childhood is operationalized to three sub-dimensions including economic conditions, community environment, and peer network.

## 3. Study Design

### 3.1. Theoretical Framework

The direct mechanism employed in this study shows that childhood experience has a direct impact on the mental health of the elderly. Firstly, economic conditions during the childhood being a core factor to mental health in later years have been well documented in previous literature [6,22,23]. Secondly, the community environment is another important factor to health [35]. Sterling [36] found that people living in communities with close neighborhood relations and sound reputation are in a better mental state than those living in communities with bad neighbors and a high crime rate. Thirdly, Li [37] revealed the important role of a good peer network in avoiding health risks. Stevenson [38] thought that a high-quality peer network and its interaction can alleviate the negative impact of the surrounding social environment on mental health. It can be seen that the economic conditions during childhood, the environment in which children live, and the peer network they contact, all have important influences on the mental health of older people.

**Figure 1:**
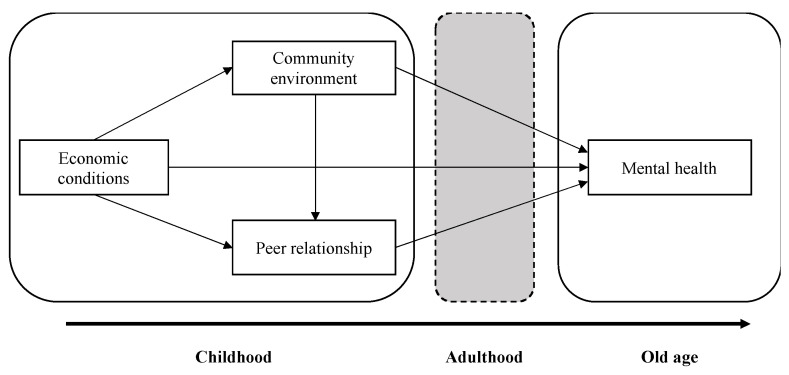
Path model of the relationships between earlier experience and later mental health.

As to the relationships among those three factors, the level of the family economy determines the quality of children’s living environment and peer network to a certain extent [39]. In other words, on the one hand, the economic conditions in childhood can be regarded as the primary factor affecting the mental health of the elderly, the peer relationship quality is both dependant on the family economic conditions and their community environment on the other hand. However, the mediating mechanism of adulthood as the indirect way claimed is excluded from current study. Thus, we construct a path model of the relationships between the life experiences during childhood and the mental health in old age as shown in Figure 1.

### 3.2. Data Sources

Data used in this paper are from the China Health and Retirement Longitudinal Study (CHARLS), which is a national longitudinal survey hosted by the National Development Research Institute of Peking University with the aim of better understanding the socioeconomic determinants and consequences of ageing. The baseline survey was conducted in 2011 followed by wave 2 (2013), wave 3 (2015), and wave 4 (2018) accompanied by a special life course one in 2014. As many as 25,000 individuals in 12,400 households were interviewed covering 28 out of 31 provinces, 450 communities. Its high representativeness and quality enable us to identify the factors to older people’s mental health from a life course perspective.

In this study, we combine the data from the special survey in 2014 and wave 3. Information referring to the life experiences during childhood is derived from the life course survey. Those related to the demographic information and mental health are from wave 3. There are 20,654 and 21,095 samples in above two of the waves respectively and 18,608 individuals were interviewed in both surveys. After excluding the sample younger than 45 years old and those with missing values of the dependent variable, we got 9750 valid samples.

### 3.3. Variable Measurement

Dependent variable: mental health of older people. The mental health index (MHI) was generated by summing the scores of 10 questions derived from the Center for Epidemiologic Studies Depression (CES-D) Scale, which is a short self-report scale designed to measure depressive symptomatology in the general population [40]. This well-accepted index is also applied universally in studies on older adult populations [41].

Independent variables: childhood circumstances. Given the limited information of a single indicator, the independent variable in this study is a latent variable including three subvariables: economic conditions, community environment, and peer relationship. Each subvariable is a subjective and multidimensional latent variable.

Generally, income is the most direct indicator of economic conditions, while economic income is not yet available in childhood. We replace it with the economic conditions of the family (or parents). If a family has higher socioeconomic conditions, the chance of frequent hunger during childhood is relatively low [42]. China experienced a national natural disaster during 1959 and 1961 which resulted in as many as 5 million unnatural deaths due to starvation [43]. Since most older people in this survey were children between 1958–1962, we operationalize the economic conditions into three binary indicators related to the natural disaster: starvation experience (SE), experience of fleeing from famine (FF), and experience of relatives’ death from starvation (DS). The CHARLS survey used in this paper defines a child as one aged 17 and below. For ensuring all samples were children in the natural disaster, individuals aged 17 and below between 1958 and 1962 were selected. The rest valid sample is 9750.

The community environment variable is measured by four indicators rated on a four-point scale: safety being out alone at night in the neighborhood (SN), neighbors in the community helping each other (NH), harmony among neighbors (HN), cleanliness of the community (CC). The variable of the peer relationship is measured by three indicators rated on a four-point scale: loneliness due to the lack of friends (LF), a group of friends (GF), being bullied by kids (BK).

Control variables: referring to previous studies, gender, age, marital status, education, income in old age, residence status (*hukou*) in old age, and health status during childhood are used as control variables [6].

### 3.4. Methods

Since those three independent variables are latent ones, the structural equation model (SEM) is employed in this study. The SEM consists of a measurement model, which is a confirmatory factor analysis (CFA) between observable indicators and latent ones, and a structural model reflecting the relationships between the latent variables and the dependent one. Among them, the matrix equation of the measurement model is as follows:

(1) $$Y=\Lambda_{ｙ}\eta +\varepsilon$$

(2) $$X=\Lambda_{\chi }\xi +\delta$$

Formula (1) is a measurement model for the endogenous mediating latent variables. The vector Y includes seven observable variables to measure the endogenous latent variables community environment (η1) and peer relationship (η2). Of those, η1 is measured by four observable variables: y1(SN), y2(NH), y3(HN), and y4(CC); while η2 is measured by three explicit variables: y5(LF), y6(GF), and y7(BK). $\Lambda_{ｙ}$ denotes the factor loadings between the explicit variables (y1-y7) and the latent variables (η1-η2), and ε is the measurement error. The vector X includes three observable variables to measure exogenous latent variable: economic conditions (ξ).

The below Formulas (3)–(5) is the structural model:

(3) $$\eta_{1}=\gamma_{11}\xi_{1}+\zeta_{1}$$

(4) $$\eta_{2}=\beta_{21}\eta_{1}+\gamma_{21}\xi_{1}+\zeta_{2}$$

(5) $$Y=\beta_{31}\eta_{1}+\beta_{32}\eta_{2}+\gamma_{31}\xi_{1}+\zeta_{4}$$

where $\gamma_{11}$ is the coefficient matrix between community environment (η_1_) and economic conditions (ξ_1_), $\beta_{21}$ is the coefficient matrix between community environment (η_1_) and peer relationship (η_2_), other coefficients are the similar means. ζ is the prediction error of the each structural model. Y is the dependent variable mental health which is an observable one measured by the CES-D.

## 4. Empirical Results

### 4.1. Descriptive Analysis

The specific definitions and descriptive statistics of the variables are shown in Table 1. It can be seen that the average age of the respondents is 63 years old; the gender ratio of men and women is balanced; the majority of the respondents are married; the income level of the elderly is low. The average mental health score of the elderly in China is 32 (out of 40), indicating a middle to high health status.

**Figure 2:**
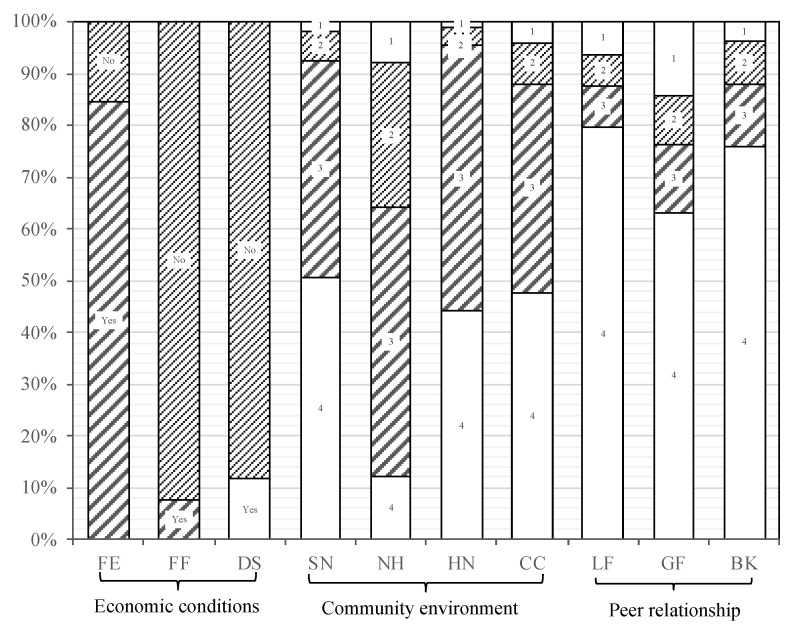
Column chart of ten indicator variables.

As for the three latent variables of life experiences during childhood, the differences within groups referring to the economic conditions are extremely large, while the means of the other variables are moderate. To visualize the distribution of the answers for each measure, Figure 2 shows column charts for the 10 categorical measure variables. Eighty-five percent of the elderly have endured hunger themselves or their family members, while the proportions of those who have experienced their family members fleeing or dying of hunger are significantly smaller, which reflects the poor economic situation of the elderly during their childhood. Although the majority of older people have experienced starvation when they were children, few fled from famine and died of hunger. In terms of community environment, the mean values of each indicator are all basically around 3.3 (out of 4), with about 90 percent of respondents living in communities with high safety, harmonious neighborhood relationships, and clean community environments. As for peer relationship, only 18 percent of the respondents had ever felt lonely because they had no friends, about 85 percent often played with good friends when they were children, and 86 percent had never been bullied by neighborhood children.

### 4.2. Base Model Estimation Results

We analyze the relationships among the variables with the help of SEM in the statistical analysis software Mplus 8.0, and the maximum likelihood estimation method (ML) is used due to its robust estimation even if the measured indicators do not obey normal distribution under the premise of large samples [44]. On the measurement models, the coefficients of all measurement indicators explaining the latent variables are significant at the 1% level, indicating that the indicators measured the latent variables better. In terms of model fit, RMSEA = 0.035 (<0.05), CFI = 0.940 (>0.9), and TLI = 0.915 (>0.9), all of these parameters meet the requirements for the fitting results of the structural equation. Furthermore, the above results remain significant after adding the control variables such as gender, age, and income, which is similar to the above one, thus the model can be considered to fit well and the results are satisfactory.

The structural model in Table 2 presents the estimated results between economic conditions, community environment, peer relationship, and mental health. Given the purpose of this study, the control variables are not reported in the following tables. Results suggest that economic conditions and peer relationship have positive direct contributions to the mental health of older adults. The standard path coefficients of them are 0.094 and 0.316, respectively, with significance at the 1% level, which indicates that for every one-unit increase in economic conditions and peer network during childhood, the mental health of older adults is improved by 9.4 percent and 31.6 percent. It can be seen that peer relationship in childhood has a stronger impact on the mental health of older people. The result of the effect of economic conditions in childhood on older adults’ health is consistent with existing studies [23], which paid less attention to the effect of children’s peer network on health in later life. The path coefficient of community environment on mental health was −0.037 but without statistical significance, indicating that the community environment in childhood did not directly affect the mental health of the elderly. The effect of the community environment on mental health is completely mediated by the peer relationship.

Table 3 shows the affecting mechanism linking the earlier experience in childhood and mental health in later life. Since the direct effect of residential environment is not statistically significant, the result of this pathway was not reported in the table. Economic conditions in childhood have both a direct positive effect (0.094) and an indirect positive effect on mental health in later life, with the mediating effects of the peer relationship and the community environment: economic conditions → peer relationship → mental health (0.363 *0.316 = 0.115); economic conditions → community environment → peer relationship → mental health (0.187 *0.520 * 0.316 = 0.031). The total effect coefficient of economic conditions on mental health was 0.24. Community environment in childhood had only an indirect positive effect on mental health in later life, namely, community environment → peer relationship → mental health (0.164). Peer relationship plays a crucial role in the mental health of older people as a mediating variable of economic conditions and community environment. Thus, peer relationship in childhood has the greatest effect on the mental health of older adults, followed by economic conditions and finally, community environment.

### 4.3. Multiple-Group Comparisons

#### 4.3.1. Age Cohorts

Older people are a large age range group, and the health of this group is quite different. The health of the older population declines as they age. For example, the prevalence of disability is much higher in the oldest [45]. In this paper, when analyzing the mental health index of the elderly, we also found that there is a large variation in the mental health of the older people, with about 36 percent of the elderly having a mental score of 35 and above (out of 40), but still also, 7.6 percent having a score of less than 20, which shows that the health of the elderly is inequal. Therefore, this paper must analyze the age difference of mental health of older people with multiple comparisons.

Since the upper and lower bounds of age are 54 and 74, respectively, after removing the missing value samples of the explanatory variables, we use the 54–59 age group as the first group, and after that, the elderly are divided into five groups by age with an interval of 5 years. There are four groups, as seen in Table 4.

First of all, the result confirms the heteroscedasticity of health status among age groups. As can be seen from the estimates in Table 4, economic conditions have a significant effect on mental health only for the younger older people aged 64 and below with a graduate decrease. It is the same as the facilitative effect of economic conditions on peer relationship and community environment. This suggests that the role of economic conditions in childhood on the mental health of older people falls with age, but can still have some impacts on mental health for the younger.

Second, community environment does not have a direct effect on the mental health of older people in all four age groups as it does in the baseline model, but it plays an indirect role through peer relationship. Specifically, community environment has a positive effect on peer relationship in all four age groups with a statistical significance at the level of 1 percent. This finding suggests that the mental health of older adults indirectly benefits from a good community environment during childhood.

Third, the effect of peer relationship on mental health is significantly positive at the level of 1% for all ages with an increasing trend with age. This is a strong indication of the long-term effect of peer relationship on the mental health of older people.

#### 4.3.2. Groups by Gender

Health differences by gender are prevalent in reality. Women are at a health disadvantage compared to men in all ages derived for a variety of factors, such as the lower health returns from education and income [46]. A possible explanation may be that the traditional gender role norms have significantly different role expectations on division of labor for men and women. Not only do the types of work differ, but men are able to get more employment and promotion opportunities and are more competitive in the market, while women have to take care of their families and undertake domestic work in addition to the labor market. Those gender varieties in labor division may influence the mental health [47]. Thus, we also analyze gender heterogeneity in the mental health of older people, as shown in Table 5.

The economic conditions in childhood have a significant direct effect on the mental health of older women, which is not significant for men. In addition, the contribution of economic conditions to peer relationship and community environment is greater for men than for women, suggesting that men with better economic conditions in childhood are more likely to live in a better community or have good peer relationships. The community environment equally effects the mental health for both genders. However, the effect of community environment on women’s peer relationship is significantly greater than men’s, which means that women living in better communities are more likely to have good peer relationships than men. This finding reflects that men’s good peer relationship depends mainly on better economic condition, while women’s is more determined by better community environment.

## 5. Discussion

Although we have made huge advances in life expectance over the last century, the extent of the opportunities that arise from increasing longevity will be heavily dependent on the health of these older populations. Unfortunately, there is little evidence to indicate that older people today are experiencing better health than their parents did at the same age [48]. Moreover, good health in old age is not equally distributed, either between or within countries. Health is a systematic result affected by a diverse range of social, economic, and environmental factors. Increasingly, the life course approach is well accepted by researchers recently to understand population health and well-being, especially for older adults. This perspective treats health as a cumulative product of risk behaviors, protective factors, and environmental agents that we experience throughout our whole lives. For example, the gradual accumulation of risk models encourages researchers to focus on how risk factors at the earlier life stage combine to raise disease risk. In this study, the life course perspective is used to empirically investigate the direct effect of life experiences during childhood on mental health in old age based on a national longitudinal survey in China. Compared to previous studies, it is the first time in China that a model with multiple aspects of life experiences and their interaction as defined mostly by life course theory is considered in a whole empirical model. We are aimed at discussing a twofold question. First, whether childhood experiences are associated with mental health in old age? If so, then what mechanisms may be responsible for those associations?

Compared to other life experiences, the peer relationship during childhood whereas being ignored by previous studies determines to a large extent the mental health through the whole life course especially in old age. A great deal of studies have examined the association between peer relationship and the mental health for a particular age cohort such as adolescents, working-age adults [49]. In this study, we confirmed a significantly larger “long arm” effect of the interpersonal relationship in childhood than the economic conditions and community environment on later mental health both in the baseline model and multiple-group comparison ones. The interpersonal relationship is a displayer of the individual’s personality which is hardly changed once formulated in childhood as the Chinese proverb says that ‘you may figure out a person’s future from his childhood’. A child with an outgoing personality would be glad to make more friends which will in return reinforce his/her characters. This cumulative effect is less mediated by the changed life environments in the adult stage. Moreover, we identify a progressive effect of the interpersonal relationship on the oldest old in the age cohort comparison model in which the coefficient significantly increases from 0.251 in the younger group aged between 54 and 59 to 0.462 in the oldest one aged 70 and above. While this difference between men and women is not founded.

Although the effect of the family economic conditions is confirmed in our models, their influence force is not as strong as described in previous literature. The direct effect coefficient is less than 0.1 compared with 0.32 in terms of the peer relationship. Benefited from the mediating effect of the peer network, the indirect effect of the economic conditions reaches 0.15 and the total effect is 0.24 which is still less than that of the peer network. This result may be attributed to the indirect mechanism in which the effect of the economic conditions during childhood is mediated by the improved life conditions in adult age [25]. In other words, contrary to peer relationship, impact of the economic conditions on mental health in old age is at greater risk of being mediated by later life experiences. This inference is examined by the multi-group comparison model by age cohort in which the coefficient of the economic conditions on mental health in Table 4 decreases gradually with the age growth and the statistical significance is significant at the 5% confidence level for the younger elderly aged 64 and below while it disappears for those aged 65 and above. The multi-group comparison by gender suggests that the direct impact of the economic conditions on mental health in old age is confined to women. Unlike previous literature, the direct impact of the community environment on older people’s mental health is not found in all models. Socio-ecological theory argues that community socioeconomic conditions play a vital role in determining variation in individuals’ health [30,50]. This theory incorporates a set of various indicators including both macro and micro aspects aimed at health promoting interventions. As for the community environment related to neighborhood relationships measured by the current research, children are unable to be aware of the community relationships among adults.

Despite the discrete associations between the childhood circumstances and mental health in old age, we find a distinct interaction mechanism linking those two variables. As a core mediating variable, the peer relationship bridges the economic conditions, community environment and the mental health of older adults. The better economic family and community environment a child lives in, the closer fellowship he/she has, which then positively contributes to the better health. This finding replenishes the growing body of life course literature on health determinations. Different from the previous studies viewing all aspects of the socioeconomic conditions equally, our model results suggest that factors to older people’s mental health are unequal. Those related to economic and material conditions during childhood are changeable and apt to indirectly effect the mental health in old age, while the intimate peer relations in childhood contribute to their senses of belonging and mental health which is permanently active throughout the whole life course as called ‘the long arm of childhood’ by Harward and Gorman [51].

Our findings bring into sharp focus the idea that social policies aimed at promoting older people’s mental health in the context of active ageing and healthy ageing strategy should go beyond the old age stage and target at improving interpersonal partnerships in early childhood, accompanied by social policies related to socioeconomic conditions including family policies on improving the material living conditions of families with children, community safety, especially with the focus on children’s inclusion/exclusion. Our research recommendations are indeed aimed more at people who are now children, but it is not meaningless for those who are already old. As mentioned in the “Multisectional action for a life course approach to healthy ageing: draft global strategy and plan of action on ageing and health”, when we recognize that many diseases in older adults are preventable, we can prevent them at younger ages by promoting physical activity. This means that for relatively younger older adults, we can still uphold the concept of prevention and intervene in their lives to achieve better health outcomes. The Chinese economy has made outstanding strides, accompanied by a sharp increase in inequality and an entire overlook in social construction. Although the overall nutritional status of children has improved considerably, the prevalence of malnutrition among children in less developed areas is still maintained at a high level. Thus, a promising policy on mental health based on life course in China should give more weights to the disadvantaged social groups with all-round supports.

## 6. Conclusions

Our study illustrates the multifarious ways in which childhood circumstances are tied to mental health in old age using a nationally representative longitudinal survey in China. We compare the unequal impacts of childhood living conditions on mental health in old age and obtain some valuable conclusions being different from previous literature. This study highlights the importance of peer relationship among children to their mental health in later life and its mediating effect on associations between family economic conditions as well as community environment and later mental health. Those findings not only enrich the life course theory, but also give policy lessons to policymakers who devote their efforts to promote citizens’ health status including older people, especially in terms of developing countries where social policies targeting families with children are scarce and socioeconomic inequality is severe.

Limited by the data availability, there are several limitations in this article. The most meriting discussion is that we are unable to map an unabridged trajectory of the health function as theoretically illustrated by the life course paradigm. We just focus on the direct mechanism linking the childhood circulations and mental health in old age while the indirect one is not included into consideration. The indirect mechanism pays most attention to the experience in adulthood to identify its mediating effect between the childhood and older age stages. If doing so, more research will uncover further details on how childhood circumstances are tied to mental health in older age. In addition, childhood measures used in this paper were retrospectively reported, which may imply a response bias. 

## Figures and Tables

**Table 1:** Descriptive statistics (N = 9750).

	Variables	Value Description	Mean	SD	Min	Max
**MHI**		Numerical variable	31.83	6.59	10	40
Economic Conditions	FE	1 = No; 0 = Yes	0.15	0.36	0	1
	FF	1 = No; 0 = Yes	0.92	0.27	0	1
	DS	1 = No; 0 = Yes	0.89	0.32	0	1
Community Environment	SN	1 = Not safe at all; 4 = Very safe	3.41	0.69	1	4
	NH	1 = Not willing to at all; 4 = Very willing to	3.31	0.79	1	4
	HN	1 = Not close-knit at all; 4 = Very close-knit	3.38	0.61	1	4
	CC	1 = Not clean at all; 4 = Very clean	2.68	0.79	1	4
Peer Relationship	LF	1 = Often; 4 = Never	3.61	0.86	1	4
	GF	1 = Never; 4 = Often	3.25	1.11	1	4
	BK	1 = Never; 4 = Often	3.60	0.80	1	4
Control Variables	Gender	1 = Male; 0 = Female	0.50	0.50	0	1
	Age	Numerical variable	62.99	5.43	54	74
	Health status	1 = Healthy; 0 = Unhealthy	0.36	0.48	0	1
	Hukou	1 = Rural; 0 = Urban	0.23	0.42	0	1
	Marital status	1 = Married; 0 = Unmarried	0.99	0.09	0	1
	Education	1 = High school and above; 0 = Below middle school	0.14	0.35	0	1
	Log of income	Numerical variable	6.66	2.94	0.69	11.25

Notes: The full name of the abbreviation of the measured variables are shown in parentheses. FE (starvation experience), FF (experience of flee from famine), DS (experience of relatives’ death from starvation), SN (safety being out alone at night in the neighborhood), NH (neighbors in the community helping each other), HN (harmony among neighbors), CC (cleanliness of the community), LF (loneliness due to the lack of friends), GF (a group of friends), and BK (being bullied by kids).

**Table 2:** Model maximum likelihood estimation results.

Model		Coefficients	S.E.	P
**Structural Model**				
Economic Conditions → Community Environment		0.187	0.025	**
Economic Conditions → Peer Relationship		0.363	0.035	***
Community Environment → Peer Relationship		0.520	0.024	***
Economic Conditions → Mental Health		0.094	0.030	***
Community Environment → Mental Health		−0.037	0.027	0.177
Peer Relationship → Mental Health		0.316	0.038	***
**Measurement Model**				
Economic Conditions	FE	0.256	0.020	***
	FF	0.214	0.020	***
	DS	0.487	0.030	***
Community Environment	SN	0.371	0.012	***
	HN	0.716	0.010	***
	NH	0.740	0.010	***
	CC	0.220	0.013	***
Peer Relationship	LF	0.435	0.017	***
	GF	0.372	0.015	***
	BK	0.364	0.015	***
**Goodness-of-Fit Indices**				
RMSEA = 0.035; CFI = 0.940; TLI = 0.915; χ^2^ = 6338.974				

Note: *** and ** represent 1% and 5% significance, respectively.

**Table 3:** Direct, indirect, and total effects of the model.

Factors	Paths	Coefficients
Economic Conditions	Direct effect	0.094
	Indirect effect	0.146
	Total effect	0.240
Community Environment	Indirect effect/Total effect	0.164
Peer Relationship	Direct effect/Total effect	0.316

**Table 4:** Multi-group comparison by age cohort.

Age Cohort	54–59	60–64	65–69	70+
Economic conditions → mental health	0.154(0.05) **	0.131(0.06) **	0.037(0.04)	0.049(0.07)
Community environment → mental health	0.015(0.04)	−0.012(0.05)	−0.069(0.05)	−0.100(0.09)
Peer relationship → mental health	0.251(0.06) ***	0.201(0.08) ***	0.393(0.06) ***	0.462(0.11) ***
Economic conditions → peer relationship	0.410(0.06) ***	0.402(0.07) ***	0.231(0.06) ***	0.309(0.08) ***
Economic conditions → community environment	0.148(0.05) **	0.253(0.05) ***	0.145(0.04) ***	0.090(0.05)
Community environment → peer relationship	0.480(0.04) ***	0.516(0.05) ***	0.558(0.04) ***	0.574(0.06) ***
N	3564	3112	2274	1430

Note: *** and ** represent 1% and 5% significance, respectively.

**Table 5:** Multi-group comparison by gender.

Gender Cohort	Men	Women
Economic conditions → mental health	0.070(0.043)	0.122(0.042) **
Community environment → mental health	−0.044(−0.044)	−0.100(0.045)
Peer relationship → mental health	0.359(0.049) ***	0.316(0.060) ***
Economic conditions → peer relationship	0.389(0.049) ***	0.338(0.051) ***
Economic conditions → community environment	0.228(0.035) ***	0.150(0.035) ***
Community environment → peer relationship	0.458(0.033) ***	0.577(0.035) ***
N	4915	4835

Note: *** and ** represent 1% and 5% significance, respectively.

## Data Availability

Restrictions apply to the availability of these data. Data is obtained from China Health and Retirement Longitudinal Study and are available at http://charls.pku.edu.cn/pages/data/111/zh-cn.html (accessed on 14 June 2021) with the permission of China Health and Retirement Longitudinal Study.
